# *Chlamydia trachomatis* Infection in HIV-Infected Women: Need for Screening by a Sensitive and Specific Test

**DOI:** 10.1155/2013/960769

**Published:** 2013-12-05

**Authors:** Sonali Bhattar, Preena Bhalla, Sanjim Chadha, Reva Tripathi, Ravinder Kaur, Kabir Sardana

**Affiliations:** ^1^Department of Microbiology, Maulana Azad Medical College, Delhi University, Bahadur Shah Zafar Marg, New Delhi 110002, India; ^2^Department of Obstetrics & Gynaecology, Maulana Azad Medical College, Delhi University, New Delhi 110002, India; ^3^Department of Dermatology & STDs, Maulana Azad Medical College, Delhi University, New Delhi 110002, India

## Abstract

Reproductive tract infection (RTIs)/sexually transmitted infections (STIs) are recognized as a major public health problem, particularly due to their relationship with HIV infection. Early detection and treatment of *Chlamydia trachomatis* infection (CTI) among HIV-infected and HIV-uninfected women may impact heterosexual HIV transmission. A total of 120 participants were enrolled: 30 HIV seropositive women with symptoms of RTIs, 30 HIV seropositive women without symptoms of RTIs, 30 HIV seronegative women with symptoms of RTIs, and 30 HIV seronegative women without symptoms of RTIs. One endocervical swab was collected from all participants and CTI was detected by real-time PCR (COBAS TaqMan CT Test, v2.0). CTI was detected in 4 (6.67%) HIV-infected women and in 1 (1.67%) HIV-uninfected woman (OR 4.214; 95% CI 0.457–38.865). Vaginal discharge was present in almost half of HIV-infected and HIV-uninfected women; lower abdominal pain was present in 11 (18.3%) of HIV-infected and in 9 (15%) of HIV-uninfected women. This study showed that CTI is more prevalent among HIV-infected females as compared to HIV-uninfected females. As the use of real-time PCR is not feasible in most hospitals, efforts should be made to develop a simple, sensitive, and specific test to identify women with CTI for prevention of sequelae and HIV transmission.

## 1. Introduction

Genital infection due to *Chlamydia trachomatis* is one of the most prevalent bacterial sexually transmitted infections (STIs) [1]. According to the WHO estimates, globally 92 million new cases of *C. trachomatis* infection occur each year and about two-thirds of these cases occur in the developing world, where diagnostic and treatment services are scarce [2, 3]. Most epidemiological data on *Chlamydia trachomatis* infection (CTI) is from industrialized nations and reliable data from the resource poor developing nations is not available where the disease burden is concentrated. However, it is important to document laboratory-confirmed incidence and prevalence of CTI from the developing world as well. The available Indian data show a wide variation in CT prevalence with infection rates in Indian women ranging from 3.3% to 33% depending on the population sampled [4–13].

Infection with this agent is usually asymptomatic in up to 80% of women which makes diagnosis and detection all the more difficult. Left undetected and untreated the infection may evolve into pelvic inflammatory disease and may result in serious sequelae, such as ectopic pregnancy and infertility [14, 15]. CTI in women has also been linked to adverse pregnancy outcomes like recurrent miscarriage and preterm labor and may cause conjunctivitis, nasopharyngitis, and pneumonia in newborns by vertical transmission [3]. Because the infection is easily treatable with antibiotics, early detection and treatment of infected individuals are the key to prevent adverse sequelae among those infected and reduce *C. trachomatis *transmission [16]. Thus, it is important to screen adolescents and sexually active women for CTI even if they are asymptomatic [17, 18]. But, in the developing countries with the exception of sporadic testing, screening for *Chlamydia* is rarely done.

Epidemiological studies have also shown that untreated genital *Chlamydia* infection can lead to an increased risk for heterosexual acquisition of HIV. Hence, screening for CTI done in high risk populations can assist in designing HIV risk reduction strategies [8]. On the other hand, immunosuppression due to HIV may lead to more aggressive *Chlamydia* disease conditions like PID in HIV seropositive women. Thus, screening for CTI in HIV seropositive women is highly recommended to prevent morbidity associated with the disease and devastating clinical consequences [19].

Different diagnostic modalities for detection of CTI like serology, culture method, ELISA for antigen and antibody, direct fluorescence assay (DFA) and nucleic acid amplification tests (NAATs) have been used in the last 20 years but none of them are 100% sensitive. Each has its own limitations [14].  Table 1 shows the prevalence of CTI detected by using different diagnostic techniques in New Delhi population.

Polymerase chain reaction is an accurate, rapid, and reliable method for the detection of *Chlamydia trachomatis* [25]. Real-time PCR has increasingly been used and is easier to perform and faster, and since it is performed in a closed system it is less prone to contamination than the conventional PCR [26]. Keeping the above background in mind this study was undertaken to generate reliable data regarding prevalence of CTI in HIV-infected and HIV-uninfected women by real-time PCR, the most sensitive and specific test available currently for diagnosing genital *Chlamydia* infection. The primary objective of this study was to establish the need for screening HIV seropositive women for CTI.

## 2. Materials and Methods

### 2.1. Study Population

Study subjects were recruited as follows:

- thirty adult HIV seropositive women with symptoms suggestive of RTIs (study group A1),
- thirty adult HIV seropositive women without symptoms suggestive of RTIs (study group A2),
- thirty age and sex matched adult HIV seronegative women with symptoms suggestive of RTIs (control group B1),
- thirty age and sex matched adult HIV seronegative women without symptoms suggestive of RTIs (control group B2).

All the study subjects were enrolled from the Integrated Counseling and Testing Center (ICTC) for HIV/AIDS, Department of Microbiology, Maulana Azad Medical College, which is attached to the Lok Nayak Hospital, New Delhi.

### 2.2. Study Design

This study was conducted prospectively between July 2010 and January 2011 at the HIV molecular laboratory of the Department of Microbiology, Maulana Azad Medical College. This was a cross-sectional analysis to determine the prevalence of *Chlamydia trachomatis* infection by using real-time PCR in HIV seropositive and seronegative, symptomatic, and asymptomatic women visiting the ICTC of our department. Subjects were enrolled in this study following institutional ethical committee clearance and a written informed consent of all participants. Each participant was interviewed using a questionnaire concerning general sociodemographic information, personal details, and clinical symptoms. Subjects having one or more of the following symptoms were considered symptomatic for RTI [27]:

- vaginal discharge,
- vesicular and/or non­vesicular genital ulcers,
- inguinal bubo,
- lower abdominal pain,
- genital skin conditions,
- urinary burning or frequency,
- dysmenorrhea, menorrhagia, and intermenstrual bleeding.

Each study subject then underwent a general physical, per abdomen, per speculum, and per vaginum examination.

### 2.3. Laboratory Methods

Diagnosis of HIV infection was done by following the standard protocol at our ICTC that employs pretest and posttest counseling and obtains informed consent before HIV testing. Three different rapid tests were used to detect HIV-1 and HIV-2 antibodies (COMBAIDS (Span Diagnostics Ltd.), Retrocheck HIV (Qualpro Diagnostics), and Tri-line (Rapid Diagnostics)) following the manufacturer's instructions.

One endocervical swab was collected from all participants according to the instructions provided in the specimen collection and transport kit (AMPLICOR STD swab collection and transport set) for detection of *Chlamydia trachomatis* by real-time PCR. Genital *C. trachomatis* infection was diagnosed by using COBAS TaqMan CT Test, v2.0, an *in vitro *nucleic acid amplification test for the qualitative detection of *Chlamydia trachomatis* DNA in female endocervical swab specimens. Specimens were processed using the AMPLICOR CT/NG specimen preparation kit for manual specimen preparation and the COBAS TaqMan 48 analyzer for automated amplification and detection (Roche Diagnostics). The test was performed and reading was interpreted as per the kit literature.

### 2.4. Statistical Analysis

95% confidence interval was tested to ascertain whether the results were statistically significant. Odd ratio was calculated for relative risk determination.

## 3. Results

A total of 120 participants were enrolled for this study. The age and gender profile of all the participants is shown in Table 2. In both the HIV seropositive study group and the HIV seronegative control group the age group of 26–35 years was the predominant age group. The mean age of HIV-infected cases was found to be 30.92 ± 5.7 years and in HIV-uninfected controls 28.52 ± 6.9 years.

Table 3 shows the presenting complaints of the symptomatic women in both the HIV seropositive study group and the HIV seronegative control group. Vaginal discharge and lower abdominal pain were the most common presenting complaints amongst the symptomatic participants.

Table 4 shows the STI/RTI syndromes diagnosed in study subjects on per speculum examination. Vaginitis was the most common syndrome diagnosed in both the study and the control groups.

Table 5 shows the correlation between presence of symptoms and CTI in the study and the control groups. *Chlamydia trachomatis* infection was more commonly diagnosed in the asymptomatic HIV seropositive study subjects as compared to the symptomatic HIV seropositive study subjects.

In our study the prevalence of CTI was higher in HIV seropositive women as compared to HIV seronegative women (OR 4.214; 95% CI 0.457–38.865) and among the HIV positive asymptomatic as compared to the HIV negative asymptomatic (OR 2.111; 95% CI 1.606–2.776), although the differences were not found to be statistically significant (Table 6).

## 4. Discussion

In India STIs/RTIs and HIV/AIDS are major public health problems. Incidence and prevalence data have a key role in control strategies for HIV and STIs. Comprehensive baseline information on the epidemiology of STIs is essential for the design, implementation, and monitoring of successful control programs to reduce their incidence [28]. Routine surveillance of STIs/RTIs is not carried out in our country due to the lack of laboratory diagnostic facilities, limited resources, stigma, and discrimination associated with STIs and poor attendance of STI patients, especially women, in sexually transmitted disease (STD) clinics. Taking the above facts into consideration, it is meaningful to have genuine laboratory-confirmed data on the incidence/prevalence of RTIs/STIs in India [29]. As the asymptomatic nature of STIs [30] is well known, there is a need to adopt a specific strategy for the screening of the sexually active population in India to reduce the overall rate of STIs, which would, in turn, reduce the risk of HIV infection. Our study presents an insight into the prevalence data of genital *Chlamydia* infections in HIV-infected and HIV-uninfected women visiting the ICTC of New Delhi's largest tertiary care hospital.

The mean age of the HIV seropositive subjects in our study was 30.92 ± 5.7 and that of the HIV seronegative control group was 28.52 ± 6.9. This compares well with the findings of another study done in Baroda, India, to look for the prevalence of RTIs in HIV-infected women wherein the mean age for HIV positive women was 30 and that for HIV negative women was 27 [31]. The predominant age group in the HIV positive participants in our study was 26–35 years. This association of *Chlamydia* infection with younger age is consistent with studies from other developing countries [32, 33]. This finding supports the fact that young sexually active adults should constitute a priority target group in the STI control program. However, the findings of our study cannot be generalized to all sexually active adults as the study has its limitations of a small sample size and the study participants belonging to a high risk group being recruited from the ICTC of our department. 80% of our HIV positive study participants were married which is quiet similar to what has been reported by another study from sub-Saharan Africa in which 85.3% of the HIV-infected women were married [34]. This finding highlights the importance of concurrently screening and treating spouses/sexual partners to decrease the STI burden in the country but this is challenging due to the lack of knowledge and cooperation from husbands especially in the Indian setup.

In the present study, vaginal discharge and lower abdominal pain were the 2 most frequently reported symptoms by the symptomatic women in both the study and the control groups, confirming the data reported by some previous Indian studies [22, 29]. Another notable observation of our study was that vaginitis was the most common clinical finding detected on per speculum examination in both the HIV positive (50%) and the HIV negative groups (56.67%) which is in accordance with the observations of Garg et al. and Balamurugan and Bendigeri where a majority of women on clinical examination had vaginitis of 94.6% and 36.9%%, respectively [22, 35].

The present study also validates the fact that *Chlamydia* infections are usually asymptomatic as 10% of the asymptomatic women were diagnosed with CTI while only 3.3% of symptomatic women had lab-confirmed CTI in the HIV positive study group. This finding has been documented by another study done in Canada [36]. Once again this emphasizes the importance of routine screening of at risk young sexually active women.

In our study CTI was detected in 6.67% (4/60) HIV-infected women and in 1.67% (1/60) HIV-uninfected women by real-time PCR although this difference was not statistically significant but the odds ratio was 4.214. A study from Cuba reported *C. trachomatis* infection in 10% of HIV-infected cases and 6.6% of HIV-uninfected cases by nested PCR but the difference was not statistically significant while the Odds ratio was 3.39 which is in concordance with our study [37]. Studies conducted by Natividad-Villanueva et al. in USA and Seck et al. in Senegal reported *C. trachomatis* infection in 3.33% and 2.1% of HIV-infected females, respectively [38, 39]. Larger studies are required to validate our observation as our study was restricted to a small number of cases.

## 5. Conclusions

This study clearly shows that CTI is more prevalent among HIV-infected females (with or without symptoms of RTI) as compared to HIV-uninfected females. Our study also stresses the usefulness of screening asymptomatic HIV-infected and HIV-uninfected females for CTI by risk assessment and diagnostic testing periodically to prevent the occurrence of adverse outcomes associated with the disease and also to check further spread of infection in the community. For countries like India that still do not currently have an active *Chlamydia* screening program in place, randomized controlled trials are required to delineate the benefits of screening in the sexually active population as our study participants represent a high risk group. It is also important that any test adopted in a national screening program be used in the primary care setting by practitioners without the need for expensive training and equipment. However, since use of real-time PCR is not feasible in most hospitals in developing countries efforts should be made to develop a simple, cost-effective, sensitive, and specific point of care test to identify and treat women with CTI for prevention of sequelae and HIV transmission.

## Figures and Tables

**Table 1:** Diagnostic methods for CTI.

Year	Study population	Tissue culture	DFA	ELISA for antigen	ELISA for antibody	PCR	RT PCR	Ref. no.
1993	Pregnant women	—	—	10	IgA 33.3 IgG 44.6	—	—	[20]
1999	NGU	—	—	43.8	—	—	—	[21]
2001	Community study among women	—	29.0	—	—	—	—	[22]
2004	Infant pneumonia	17.0	37.0	—	—	55.0 (in-house)	—	[23]
2005	PID and infertility	—	—	—	IgG 14 IgM 32 IgA 6.7	—	—	[24]

**Table 2:** Demographic profile of study participants.

	HIV seropositive cases (*n* = 60)	HIV seronegative controls (*n* = 60)
Age		
18–25	8 (13.3)	23 (38.3)
26–35	39 (65)	30 (50)
36–45	12 (20)	6 (10)
45–49	1 (1.6)	1 (1.6)
Marital status		
Married	48 (80)	59 (98.3)
Widowed	10 (16.6)	1 (1.67)
Divorced	2 (3.3)	0 (0)
No. of sexual partners		
1	56 (93.3)	59 (98.3)
2	3 (5)	1 (1.7)
≥3	1 (1.7)	0
Use of barrier contraception		
Yes		
Regular	4 (6.7)	6 (10)
Infrequent	8 (13.3)	16 (26.7)
No	48 (80)	38 (63.3)
History of abortion		
Yes	7 (11.6)	4 (6.6)
No	53 (88.3)	56 (93.3)

**Table 3:** Complaints among symptomatic subjects (A1 + B1) (*N* = 60).

Complaints	Study group A1 (*n* = 30) no. (%)	Control group B1 (*n* = 30) no. (%)
Vaginal discharge	28 (93.33)	27 (90)
Genital ulcer	3 (10)	1 (3.33)
Inguinal swelling	1 (3.33)	0 (0)
Lower abdominal pain	11 (36.66)	9 (30)
Genital warts	2 (6.67)	0 (0)
Itching in external genitalia	5 (16.67)	3 (10)
Intermenstrual bleeding	2 (6.67)	1 (3.33)
Urinary complaints	6 (20)	4 (13.33)
Generalized rashes	2 (6.67)	1 (3.33)
Fever	2 (6.67)	0

**Table 4:** STI/RTI syndromes among study subjects on the basis of per speculum examination (*N* = 120).

Study subjects	HIV positive study group (*n* = 60) no. (%)	HIV negative control group (*n* = 60) no. (%)
Vaginitis	30 (50)	34 (56.67)
Cervicitis	3 (5)	2 (3.33)
Vaginitis + cervicitis	3 (5)	0 (0)
Genital ulcer	2 (3.33)	1 (1.67)
Genital warts	2 (3.33)	0 (0)
NAD*	20 (33.33)	23 (38.33)

Total	60 (100)	60 (100)

*No abnormality detected.

**Table 5:** Correlation between symptoms of RTI/STI and CTI.

Symptoms of RTI/STI	HIV positive study group (*n* = 60)		HIV negative control group (*n* = 60) no. (%)	
	Present (*n* = 30) no. (%)	Absent (*n* = 30) no. (%)	Present (*n* = 30) no. (%)	Absent (*n* = 30) no. (%)
*Chlamydia trachomatis* positive	1 (3.33)	3 (10)	1 (3.33)	0 (0)

**Table 6:** Correlation between HIV positivity and presence of CTI among women with and without symptoms.

	*P* value	Odds ratio (95% CI)
HIV positive (A1 + A2) versus HIV negative (B1 + B2)	0.171	4.214 (0.457–38.865)

HIV positive symptomatic (A1) versus HIV negative symptomatic (B1)	1	1 (0.060–16.763)

HIV positive asymptomatic (A2) versus HIV negative asymptomatic (B2)	0.076	2.111 (1.606–2.776)
